# Seasonality of helminth infection in wild red deer varies between individuals and between parasite taxa

**DOI:** 10.1017/S0031182018000185

**Published:** 2018-03-09

**Authors:** Gregory F. Albery, Fiona Kenyon, Alison Morris, Sean Morris, Daniel H. Nussey, Josephine M. Pemberton

**Affiliations:** 1 Institute of Evolutionary Biology, School of Biological Sciences, University of Edinburgh, Edinburgh EH9 3JT, UK; 2 Moredun Research Institute, Pentlands Science Park, Bush Loan, Midlothian, EH26 0PZ, UK

**Keywords:** Disease ecology, Helminths, repeatability, seasonality, ungulate, wild mammal, zero-inflated models

## Abstract

Parasitism in wild mammals can vary according to myriad intrinsic and extrinsic factors, many of which vary seasonally. However, seasonal variation in parasitism is rarely studied using repeated samples from known individuals. Here we used a wild population of individually recognized red deer (*Cervus elaphus*) on the Isle of Rum to quantify seasonality and intrinsic factors affecting gastrointestinal helminth parasitism over the course of a year. We collected 1020 non-invasive faecal samples from 328 known individuals which we then analysed for propagules of three helminth taxa: strongyle nematodes, the common liver fluke *Fasciola hepatica* and the tissue nematode *Elaphostrongylus cervi*. Zero-inflated Poisson models were used to investigate how season, age and sex were associated with parasite prevalence and count intensity, while Poisson models were used to quantify individual repeatability within and between sampling seasons. Parasite intensity and prevalence varied according to all investigated factors, with opposing seasonality, age profiles and sex biases between parasite taxa. Repeatability was moderate, decreased between seasons and varied between parasites; both *F. hepatica* and *E. cervi* showed significant between-season repeatability, while strongyle nematode counts were only repeatable within-season and showed no repeatability within individuals across the year.

## Introduction

Gastrointestinal helminths include a range of nematode, trematode and cestode species and are an important selective force in wild vertebrate populations (Poulin, 2007). Although they can cause severe pathology in some cases, adult helminths can survive for years within a host; infections are commonly chronic and associated with immunosuppression and minimal overt pathology (Maizels *et al.* 2012). The life cycles of helminth parasites vary, but broadly involve the parasite living and feeding within the host and producing propagules (eggs or larvae) into the gut lumen which are shed into the environment in the faeces, from which the parasite spreads to other hosts – sometimes *via* an intermediate host (Bohm *et al.* 2007). Helminth infection is commonly quantified by counting these propagules in non-invasively collected faecal samples. Faecal egg counts (FECs) are often found to correlate well with burden (McKenna, 1981; Budischak *et al.* 2015), despite egg output being a complex product of both host and parasite biology (Sargison, 2013) which can fluctuate over time (Turner *et al.* 2010). In both livestock and wild mammals, one striking feature of FEC is their distribution, which is typically strongly right-skewed with a small number of individuals with relatively high counts (Wilson *et al.* 2004). A major challenge for helminth epidemiology is to determine the degree to which variation in FEC is driven by factors intrinsic to the host (e.g. age, sex and genotype) *vs* extrinsic factors (e.g. season, annual climate and host density). Although there is good cross-sectional evidence that FEC varies with season, age and sex in the wild, longitudinal data are essential to properly separate within- and among-individual processes, and to determine the repeatability of helminth infection within the same host. Longitudinal FEC data remain relatively scarce in wild or free-living mammals, although prior studies suggest that counts are to some degree repeatable within individuals over years and seasons (Wilson *et al.* 2004; Wood *et al.* 2013; Debeffe *et al.* 2016). Importantly, as seasonal changes can affect hosts through changes in nutrition, immunity and investment in tradeoffs (Martin *et al.* 2008), parasite intensities may differ seasonally across the host population, resulting in different individuals peaking in burden or transmission at different times of the year. It is therefore of interest to compare and contrast individual repeatability of FEC across seasons and parasite groups. We aim to use repeated counts at multiple timescales to separate real between-season differences in parasitism from baseline variation arising from experimental error or small-scale temporal fluctuations.

Parasites are well known to be influenced by seasonal extrinsic factors: weather patterns can impact parasites by affecting survival and movement of transmission stages, and regular changes in host biology such as parturition or mating seasons can affect resource availability (Altizer *et al.* 2006). In temperate regions, helminths usually show a peak in egg output during the spring and summer, with lower or arrested transmission in the winter as a result of: (i) reduced survival and mobility of parasites (Stromberg, 1997) and (ii) adaptive coincidence between infective parasites and immunologically naïve young in the spring (Wilson *et al.* 2004). However, different species do show different adaptive seasonality: for example, in Svalbard reindeer the nematode *Marshallagia marshalli* transmits throughout the winter months despite extreme cold and different host feeding patterns (Carlsson *et al.* 2012). Few comparisons of seasonality in egg counts across helminth groups have been made in wild vertebrate systems to date. Studies that rely on post-mortem sampling are often seasonally restricted to windows of mortality (e.g. Craig *et al.* 2006) or culling seasons (e.g. Irvine *et al.* 2006), and are therefore unable to investigate seasonal trends. Two important host factors associated with parasitism are age and sex. Age-dependent parasitism occurs across the animal kingdom, with higher intensities typically observed in the young and/or the elderly (Hayward *et al.* 2009). Considering gastrointestinal helminths in wild vertebrates, there is strong evidence for declining infection intensity from birth to adulthood, which is typically ascribed to two processes: first, animals gain adaptive immunity as they age (Stear and Murray, 1994; Turner and Getz, 2010); second, animals that are most susceptible to parasites and therefore show the highest egg counts are more likely to die, meaning the animals that survive to old age are those with lower parasite infections (Wilson *et al.* 2004). Increasing helminth parasitism in elderly animals has also been ascribed to immunosenescence, although the selective loss of highly infected individuals can also confound estimates of within-individual change in later life (van de Pol and Verhulst, 2006). A particular advantage of longitudinal studies is that they allow differentiation between the within- and among-individual processes responsible for age-related variation (Clutton-Brock and Sheldon, 2010). Sex differences in parasitism are another common phenomenon in wild mammal species (Poulin, 1996), with higher burdens in males than females commonly observed in polygynous mammals in particular (Moore and Wilson, 2002). This is typically linked to sexual dimorphism and increased investment in short-term reproduction rather than immunity, rendering males the ‘sicker sex’ (Zuk, 2009).

Wild ungulates are commonly infected with a range of helminth parasite species and have formed the basis of many important individual-based studies in disease ecology (Jolles and Ezenwa, 2015). Red deer have been the subject of multiple parasitological studies, due partly to their abundance in the wild where population management often requires culling (Bohm *et al.* 2007), and as farm animals, principally in New Zealand (Mason, 1994). They are of particular interest due to their ability to act as reservoirs and vectors of parasites that commonly infect livestock (Alberti *et al.* 2011; Chintoan-Uta *et al.* 2014; Davidson *et al.* 2014), and cross-sectional culling studies have formed a useful knowledge base for red deer parasitology. Previous studies of helminth parasites in wild red deer have demonstrated through cross-sectional post-mortem sampling that parasite burdens vary with age (Vicente *et al.* 2006), sex (Irvine *et al.* 2006; Vicente *et al.* 2007*a*; French *et al.* 2016), host density (Vicente *et al.* 2007*b*), supplementary feeding (Hines *et al.* 2007), testosterone level (Malo *et al.* 2009) and several measures of condition (Irvine *et al.* 2006; Vicente *et al.* 2007*a*,b). Although such cross-sectional approaches provide detailed data on the parasite community and adult parasite burden, they cannot separate within- and among-host processes involved in helminth epidemiology and host–parasite interactions. Furthermore, male deer are culled earlier in the year than females, therefore confounding sex and seasonal effects (e.g. Irvine *et al.* 2006; French *et al.* 2016). Longitudinal studies monitoring and comparing the within-host repeatability and seasonality of counts are currently lacking for helminth parasites of wild red deer, although seasonality of *E. cervi* and strongyles has been demonstrated using the collection of fresh pellets though without the full benefits of individual-level data (Vicente *et al.* 2005; Hines *et al.* 2007), and strongyles in red deer are known to undergo a season of arrested development (Connan, 1997). An earlier study examining gastrointestinal parasites of red deer living across the Isle on Rum in Scotland (Irvine *et al.* 2006) used animals culled for management purposes in the late summer–autumn (males) or autumn–winter (females). It found a high prevalence, but low burden, of strongyle nematodes *Ostertagia* spp. and *Oesophagostomum venulosum* as well as the nematodes *Nematodirus* sp., *Capillaria* sp., *Trichuris ovis*, *Elaphostrongylus cervi*, *Dictyocaulus* sp. and coccidian *Eimeria* sp. The tissue nematode *E. cervi* and the generalist liver fluke *Fasciola hepatica*, both of which have life cycles that involve an intermediate snail host, have been documented using egg counts in wild red deer (Vicente *et al.* 2007*a*; French *et al.* 2016). However, strongyle nematodes in wild red deer have rarely been studied using non-invasive methods, which is surprising given their detailed study in related livestock hosts including cattle and sheep (Hoberg *et al.* 2001). In the present study, we repeatedly collected faeces from known study individuals of different ages and sexes on Rum within and across seasons in 2016. We analysed propagule counts of the three most prevalent helminth species groups: strongyle nematodes, *E. cervi* and *F. hepatica*. Our aims were to: (i) examine the parasite fauna of the Rum red deer and identify taxa of high prevalence for statistical analysis; (ii) assess the repeatability of non-invasive parasitological measures in the deer at multiple timescales, particularly between seasons; and (iii) investigate how prevalence and intensity of infection with the abundant taxa are associated with season, host age and host sex.

## Methods

### Study area and sample collection

The study was conducted in the North block of the Isle of Rum National Nature Reserve in the Inner Hebrides, Scotland (57°N 6°20′W). The island has a mild, wet climate and the vegetation consists of a mosaic of high-quality grassland and low-quality dry and wet heath and blanket bog. The study population comprises ~350 animals at any one time. Neonates are caught during the calving period May–July and individually marked with collars, ear tags, coloured flashes and ear punches, enabling life-long individual identification. Censuses are carried out five times a month for 9 months of the year with more frequent informal monitoring between censuses allowing compilation of individual life histories. The study area population has not been culled since 1973 and runs at the carrying capacity determined by the ground and prevailing weather conditions (Clutton-Brock *et al.* 1982). Faecal sampling was conducted on a seasonal basis, with 2-week trips carried out in winter (January), spring (April), summer (August) and autumn (November); each trip was considered to be representative of the 3 month season in which it occurred. Data for this study were all collected in 2016; as red deer are born in May–June this resulted in the study sampling two different cohorts of calves, born 2015 (sampled in winter and spring before their first birthday) and 2016 (sampled in summer and autumn). Groups of individually recognized deer were observed for defecation events from 15 to 250 m using binoculars and telescopes, with the samples recovered as quickly as possible without unduly disturbing the deer. Samples were stored in ziplock bags until processing. Efforts were made to sample as many different individuals on a trip as possible, with a subset of individuals deliberately sampled more than once in each season in order to examine the within-season repeatability of parasitological measures. Faecal analysis can be affected by the hatching, development and death of parasite propagules, influenced by temperature and oxygen availability (Nielsen *et al.* 2010). For this reason, time of defecation, time of collection and date of the count were all recorded. Following a return to the field station, samples were weighed and homogenized by hand in their ziplock bags to minimize oxygen exposure. These bags were then put inside date-specific larger bags to keep them as anaerobic as possible. All samples were kept refrigerated at 4 °C until parasitological analysis. Upon return to the laboratory, the analysis was carried out within the next 8–10 weeks (see details below). Over the course of four seasons in 2016, Ns = 1020 faecal samples were collected from Ni = 328 individuals, equating to 783 different individual–season combinations with 237 within-season repeats. The sampled individuals were a mixture of calves, yearlings, 2-year olds and adults of both sexes – although adult males were sampled much less frequently (Ns = 43, Ni = 20) than adult females (Ns = 522, Ni = 137) because relatively few adult males live in the study area. The age range of animals sampled was 0–21 years old (median 3 years old).

### Parasitology

Parasitological terms will be used as defined in Margolis *et al*. (1982), with intensity based on propagule counts. ‘Burden’ refers to the number of worms of a species infecting an individual, which could not be measured directly. Strongyle FECs were carried out within 3 weeks of collection using a modified sedimentation-salt flotation method (Taylor *et al.* 2016) with an accuracy of 1 egg per gram (EPG). The method was modified from Kenyon *et al*. (2013). Briefly, 2–15 g of faecal matter was mixed with 10 mL water per gram of faeces and the mixture thoroughly homogenized to suspend the eggs. About 10 mL of this suspension was filtered through a tea strainer and washed through with 5 mL water. The resulting liquid was decanted into a 15 mL polyacrylate test tube, which was centrifuged at 1500 rpm for 2 min and the supernatant removed. The resulting pellet was mixed with the saturated salt solution and resuspended, then centrifuged again, leaving the eggs and light debris at the surface of the liquid. Using medical forceps to clamp below the meniscus, this surface layer was poured off into a cuvette which was topped up with saturated salt and then a lid was added to seal the contents. The entire surface area of the cuvette was counted at 4× magnification to give a count of eggs in 1 g faeces, revealing strongyle nematode eggs and a selection of other species the eggs of which are less dense than the salt solution. This included *Nematodirus* sp. and *Capillaria* sp. The assay also revealed oocysts of the coccidian parasite *Eimeria* sp., which came in two varieties (‘large’ and ‘small’), and segments of the cestode *Moniezia expansa*. 730 strongyle FECs were repeated to estimate the technical repeatability of this method in our hands. These counts were averaged to give an EPG value for the sample.

*Fasciola hepatica* eggs were detected through a sedimentation method (French *et al.* 2016; Taylor *et al.* 2016) conducted on 0.5–2 g of faecal matter, from the same homogenate as the strongyle FEC, within ten weeks of collection. In the interim, all samples were kept refrigerated and oxygen-deprived, both of which prevent hatching (Hurtrez-Boussès *et al.* 2001). After filtering the faecal matter through a tea strainer, the sample was left to sediment in a conical beaker for three minutes. During this time the heavier debris (including the eggs) settles to the bottom of the beaker, and the lighter material can then be removed to leave the eggs with as little debris as possible. The remaining filtrate was pipetted onto a Petri dish and stained with methylene blue (1% w/v). The Petri dish was then examined microscopically at 4× magnification and the eggs, which are yellow against blue debris background, were counted, with the counts divided by the weight in grams of the sample used.

*Elaphostrongylus cervi* and *Dictyocaulus* sp. larvae were isolated *via* a modified Baermannisation assay (Gajadhar *et al.* 1994) within four weeks of collection. 1–14 g of faecal matter was wrapped in muslin cloth and submerged fully in a 50 mL falcon tube filled with water. This was left at room temperature for 20–24 h for the L1 larvae to emerge from the faeces and fall to the bottom of the tube. The supernatant was then carefully removed to leave <2 mL containing the larvae. This fraction was preserved with Lugol's iodine and kept refrigerated at 4 °C until counting. Counts were performed on a subsample under 40× magnification and divided by the weight of faeces used to give a measure of larvae per gram.

Some samples were not large enough to be analysed for all parasite types – hence final sample sizes were 1014 (strongyles), 991 (*F. hepatica*) and 1003 (*E. cervi*). Fluctuations in faecal water content can lead to variation in per-gram FEC, particularly across host sex and age classes and across seasons (Turner *et al.* 2010). For this reason, the proportion faecal dry matter (FDM) per gram of collected faeces was calculated for each sample by drying a known weight of faecal matter in an oven at 60 °C for 48 h and then weighing the resulting solid. FDM was mean-centred around 1 to prevent changing the distribution of non-zero counts relative to the zero counts. Counts were divided by FDM to give a measure of eggs or larvae per gram of dry matter, which was rounded to the nearest whole number to allow the use of integer count-based models.

### Statistical analysis

#### Correlations of repeated counts

Since many of the parasite groups identified were present at low prevalence (<30%, see Table 1), we restricted further analyses to the three most prevalent parasite groups: strongyle nematodes, the liver fluke *F. hepatica*, and the tissue worm *Elaphostronylus cervi*. Initially, we estimated the repeatability of propagule counts at different temporal scales by calculating Spearman's rank correlation coefficients (*r*) among: (1) repeated counts from the same sample (‘technical repeatability’); (2) repeated counts from the same individual within a season (‘within-season repeatability’); and (3) averaged within-season measures from the same individual in different seasons (‘between-season repeatability’). We also investigated whether factors related to the collection and processing of the samples influenced egg counts, with simple linear models for each parasite taxon. We investigated the influence of: (a) time of collection, (b) time to processing and (c) time to counting. However, these factors had no effect and so were not included in further analysis.

**Table 1.** Seasonal prevalence and mean and maximum intensity in propagules/g faecal dry matter of each parasite found.

	Strongyles			*Fasciola hepatica*			*Elaphostrongylus cervi*			*Dictyocaulus sp.*			*Nematodirus sp.*			*Capillaria* sp.			*Eimeria* (large)			*Eimeria* (small)			*Moniezia expansa*		
	Mean Intensity	Max Intensity	Prevalence (%)	Mean Intensity	Max Intensity	Prevalence (%)	Mean Intensity	Max Intensity	Prevalence (%)	Mean Intensity	Max Intensity	Prevalence (%)	Mean Intensity	Max Intensity	Prevalence (%)	Mean Intensity	Max Intensity	Prevalence (%)	Mean Intensity	Max Intensity	Prevalence (%)	Mean Intensity	Max Intensity	Prevalence (%)	Mean Intensity	Max Intensity	Prevalence (%)
Winter	1	(7)	37	3	(47)	52	9	(118)	93	0	(0)	0	0	(3)	10	0	(4)	13	0	(7)	15	0	(8)	18	0	(0)	0
Spring	20	(157)	97	9	(155)	57	69	(299)	94	0	(7)	15	0	(4)	4	0	(11)	11	0	(18)	11	1	(34)	12	0	(44)	1
Summer	17	(167)	97	4	(50)	44	37	(337)	74	4	(193)	32	0	(8)	11	0	(11)	4	16	(930)	14	3	(145)	11	1	(65)	2
Autumn	8	(85)	64	8	(147)	62	67	(645)	76	3	(65)	36	1	(37)	18	0	(20)	4	1	(37)	8	1	(28)	9	10	(322)	7

Minimum count of every season–parasite combination was zero.

#### Intrinsic and extrinsic factors influencing parasite prevalence and intensity

To test how season, age and sex were associated with parasite egg counts and to decompose the within- and among-individual variation in these counts, we used Generalized Linear Mixed Models (GLMMs). Parasite egg count distributions are often non-normal and strongly overdispersed (Alexander, 2012) and can feature significantly more zero counts than would be expected given their distribution (Chipeta *et al.* 2014). The analysis was carried out in R version 3.4.0 (R Core Team, 2017), with the Bayesian statistical package MCMCglmm (Hadfield, 2010) which is flexible with respect to error structures. In this set of models, we used averages of any repeat measures from the same individual within a season. We fitted zero-inflated Poisson (ZIP) GLMMs, with which we generated two estimates for each fixed or random effect within the model. The first estimate (Poisson) calculated the effect a factor has on the data assuming an overdispersed Poisson distribution, including an expected number of zero counts, while the second (zero-inflation) estimated the effect that a factor has on the number of zeros in the data. We consider these two estimates as reflecting variation in parasite intensity and prevalence, respectively. We follow convention by presenting results for prevalence before intensity for each parasite taxon. This method was chosen as factors can have contrasting effects on parasite prevalence and intensity (Chipeta *et al.* 2013). The explanatory variables fitted in the models include age category (with four levels: Calf, Yearling, 2-year-old and adult), sex (female and male) and season (winter, spring, summer and autumn), with individual identity as a random effect to control for variation between individuals (Paterson and Lello, 2003). Models were run for 2.6 million iterations (thinning interval 2000, burnin 600 000). The significance of differences among factor level means was calculated by comparing the proportion overlap of the posterior distributions of the MCMC estimates for each level and then doubled to give *P*_MCMC_ following Palmer *et al*. (2017).

#### Model-derived repeatability

The variance component associated with the individual random effect within mixed effects models is often used to calculate within-individual repeatabilities (Falconer and Mackay, 1996). This method accounts for variation between individuals that occurs as a result of the model's fixed effects (i.e. originating from differences between age, sex and seasonal categories) to estimate the proportion of variation which is explained by differences between individuals. However, there is currently no accepted method of extracting repeatability from the random effects structure of zero-inflated models. We therefore re-ran our models of each parasite count on a non-zero-inflated subset of the data using a standard Poisson model featuring additive overdispersion and applied the method described by Nakagawa and Schielzeth (2010) to calculate repeatability. We included an individual identity random effect to estimate among-season variation within hosts and an individual-by-season interaction as a second random term to estimate within-season variation within hosts. In the absence of between-season repeatability, the significance of this latter term would demonstrate consistency within individuals of those repeat samples collected within seasons. Repeatabilities were calculated on the count scale rather than the latent scale. The data analysed with this model included all samples including 237 within-season repeat samples. Prior to analysis we removed the winter season data (when counts were very low for all parasite taxa) and a subset of mainly prepatent individuals (calves in the summer for *F. hepatica* and calves in the summer and autumn for *E. cervi*), as the repeatability of these counts would be of little biological interest and they were the major source of zero-inflation.

## RESULTS

### Correlations of repeated counts

**Fig. 1.**
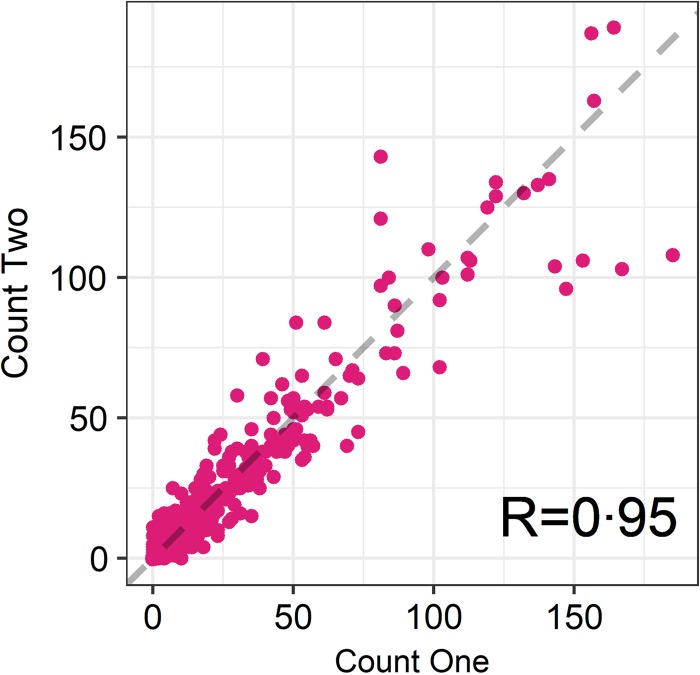
Correlation between first and second strongyle faecal egg count of the same sample (*Ns* = 730). *R* is the Spearman’s rank correlation.

**Fig. 2.**
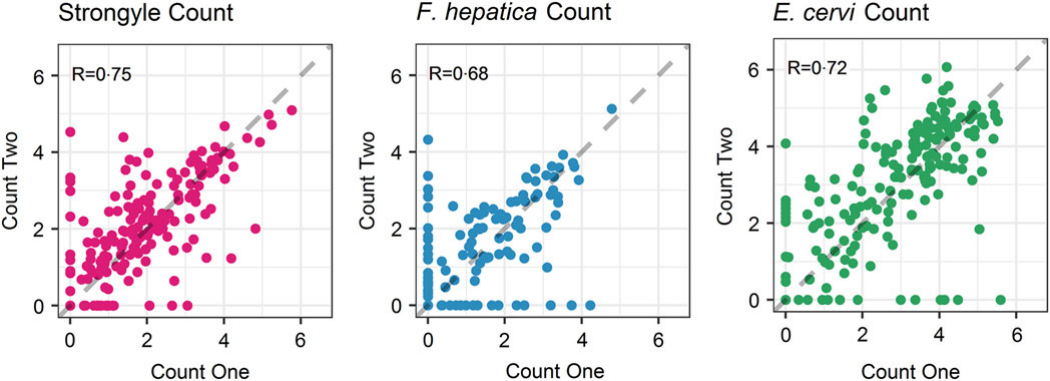
Within-season correlations of individuals’ counts from different faecal samples (A: Strongylates, B: *F*. *hepatica*, C: *E*. *cervi*). Axes have been log(*x*+1) transformed for display purposes. The dashed line represents equal counts, *y* = *x*. *R* is the Spearman’s rank correlation.

**Fig. 3.**
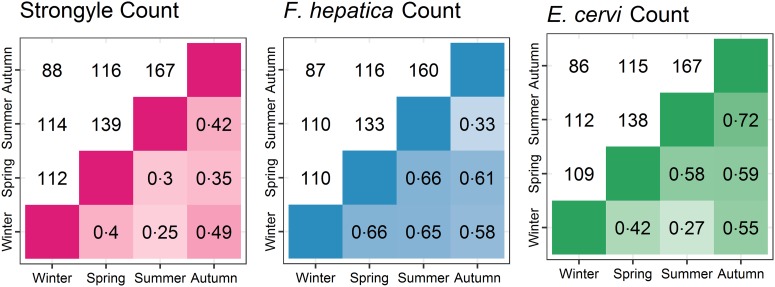
Between-season correlations of parasite counts (A: Strongyles, B: *F*. *hepatica*, C: *E*. *cervi*). Values are Spearman’s rank correlations; values above the diagonal represent the number of pairs of samples the comparisons are based on.

The seasonal prevalence, intensity and maximum count of each of the parasite taxa are displayed in Table 1. All parasites were found throughout the year except *Dictyocaulus* sp. and *M. expansa*, which were not found in the winter. Repeated strongyle counts of the same sample were strongly correlated (Spearman's *R* = 0.95, Fig. 1). Within-season resampling of the same individuals revealed strong correlations for all parasites (Spearman's *R* > 0.6, Fig. 2). Between-season averaged counts from the same individuals correlated less, and varied more between parasites (see Fig. 3 for coefficients). Strongyle nematodes showed the lowest between-season repeatability, with *E. cervi* and *F. hepatica* higher. These results were qualitatively similar to the model variance-derived repeatability estimates; see below.

### Intrinsic and seasonal correlates of counts

**Fig. 4.**
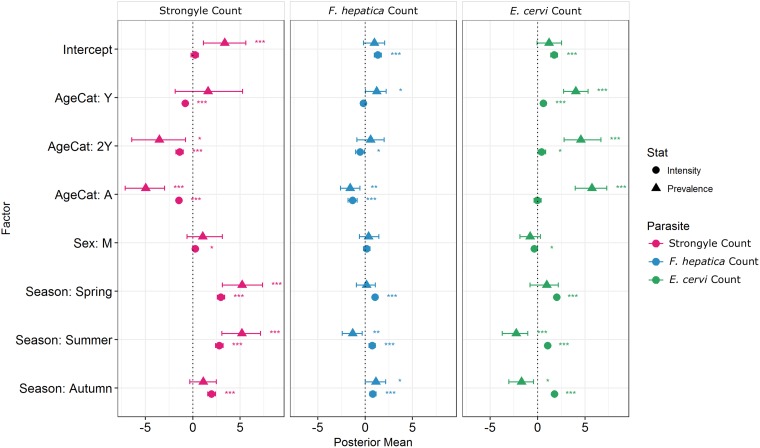
MCMCglmm zero-inflated Poisson model output for each of the three analysed parasite taxa. Points represent posterior estimates for mean effect sizes; error bars represent the 95% credibility intervals of the mean. Symbol corresponds to the statistic being estimated – zero-inflation (prevalence) or Poisson (intensity). Zero-inflation coefficients have been multiplied by −1 to aid interpretation; that is, a positive value represents a decrease in zero-inflation and therefore an increase in prevalence.

**Fig. 5.**
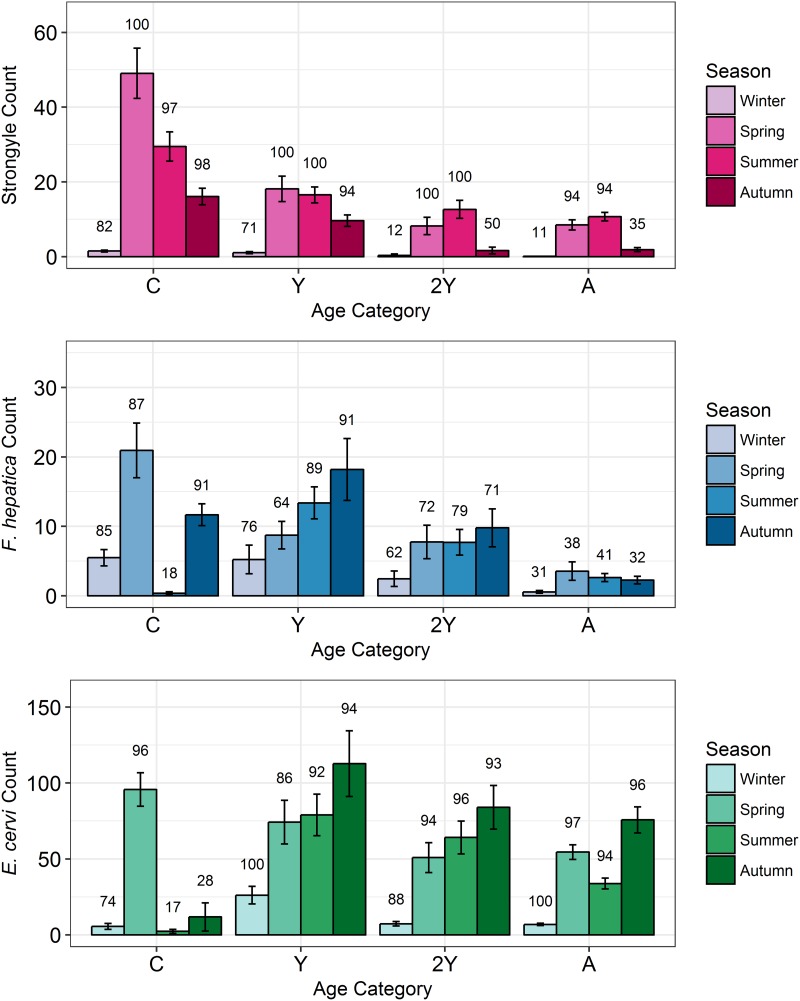
Seasonal mean intensities (±s.e.) for each parasite (A: Strongyles, B: *F*. *hepatica*, C: *E*. *cervi*) in each age category. Numbers correspond to per cent prevalence. Groups on the *x*-axis are calves, yearlings, 2-year-olds and adults in order. Figures were created using raw faecal dry matter-transformed data. The calf category represents two different cohorts: those born in 2015 (winter and spring) and those born in 2016 (summer and autumn).

**Fig. 6.**
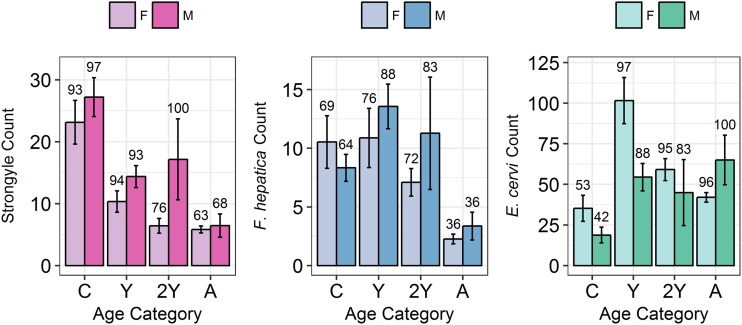
Mean infection intensity (±s.e.) for each parasite (A: Strongyles, B: *F*. *hepatica*, C: *E*. *cervi*) according to sex and age category, calculated as in Fig. 5. Numbers above the bars correspond to per cent prevalence. Groups on the *x*-axis are calves, yearlings, 2-year-olds and adults in order. Figures were created using raw faecal dry matter-transformed data.

Model results revealed contrasting trends for all three parasites according to all investigated factors (Table 2; Fig. 4). Strongyle prevalence and intensity peaked in the spring and summer (Fig. 5, *P*_MCMC_ < 0.001) and decreased in the autumn, with intensity remaining higher than winter levels (*P*_MCMC_ < 0.001) but not prevalence (*P*_MCMC_ = 0.114). Spring and summer were not significantly different in prevalence (*P*_MCMC_ = 0.988) or intensity (*P*_MCMC_ = 0.086). There was a persistent age effect in that older individuals tended to be less often infected and at lower intensities (Fig. 5). Calves showed a higher prevalence of infection than 2-year-olds (*P*_MCMC_ = 0.038) and adults (*P*_MCMC_ < 0.001) and had a higher intensity than all age categories (*P*_MCMC_ < 0.001). Yearlings also had a higher prevalence than adults (*P*_MCMC_ < 0.001), and higher intensity than both 2-year-olds and adults (*P*_MCMC_ = 0.01; *P*_MCMC_ < 0.001, respectively). Sex also had an effect in strongyles (Fig. 6), with males showing higher intensity infections (*P*_MCMC_ = 0.024) but no difference in prevalence (*P*_MCMC_ = 0.436).

**Table 2.** MCMCglmm model estimates for zero-inflated Poisson GLMMs for each analysed parasite taxon.

Factor	Statistic	Strongyles		*F. hepatica*		*E. cervi*	
		Estimate	*P*_MCMC_	Estimate	*P*_MCMC_	Estimate	*P*_MCMC_
Intercept	Prevalence	3.36 (1.14–5.59)	<0.001***	0.97 (−0.18 to 2.06)	0.082	1.2 (−0.05 to 2.54)	0.06
	Intensity	0.24 (−0.18 to 0.59)	0.232	1.34 (0.98–1.72)	<0.001***	1.77 (1.39–2.12)	<0.001***
AgeCat: Y	Prevalence	1.61 (−1.86 to 5.26)	0.396	1.22 (0.04–2.23)	0.018*	4.03 (2.76–5.32)	<0.001***
	Intensity	−0.79 (−1.02 to −0.53)	<0.001***	−0.19 (−0.46 to 0.1)	0.186	0.62 (0.35–0.92)	<0.001***
AgeCat: 2Y	Prevalence	−3.52 (−6.43 to −0.77)	0.038*	0.57 (−0.87 to 2.01)	0.466	4.57 (2.79–6.69)	<0.001***
	Intensity	−1.37 (−1.78 to −1)	<0.001***	−0.52 (−1 to −0.08)	0.024*	0.44 (0.06–0.87)	0.022*
AgeCat: A	Prevalence	−4.96 (−7.13 to −2.98)	<0.001***	−1.58 (−2.61 to −0.56)	0.002**	5.73 (3.98–7.31)	<0.001***
	Intensity	−1.45 (−1.71 to −1.18)	<0.001***	−1.31 (−1.81 to −0.85)	<0.001***	−0.03 (−0.36 to 0.38)	0.842
Sex: M	Prevalence	1.06 (−0.59 to 3.12)	0.248	0.35 (−0.6 to 1.46)	0.496	−0.8 (−1.87 to 0.31)	0.15
	Intensity	0.27 (0.05–0.52)	0.024*	0.16 (−0.19 to 0.51)	0.386	−0.33 (−0.61 to 0.01)	0.04*
Season: Spring	Prevalence	5.2 (3.12–7.35)	<0.001***	0.15 (−0.91 to 1.07)	0.782	0.97 (−0.78 to 2.18)	0.174
	Intensity	2.98 (2.6–3.37)	<0.001***	1.06 (0.79–1.34)	<0.001***	2.02 (1.82–2.22)	<0.001***
Season: Summer	Prevalence	5.18 (3.07–7.16)	<0.001***	−1.33 (−2.4 to −0.31)	0.008**	−2.26 (−3.73 to −1.02)	<0.001***
	Intensity	2.81 (2.42–3.21)	<0.001***	0.75 (0.41–1.07)	<0.001***	1.06 (0.84–1.27)	<0.001***
Season: Autumn	Prevalence	1.11 (−0.32 to 2.5)	0.114	1.12 (0.02–2.17)	0.036*	−1.7 (−3.04 to −0.42)	0.012*
	Intensity	1.98 (1.58–2.42)	<0.001***	0.81 (0.5–1.16)	<0.001***	1.78 (1.58–1.99)	<0.001***

The estimates represent the posterior mean, with 95% credibility intervals in brackets. Prevalence (zero-inflation) values have been multiplied by −1 to aid interpretation: a positive value in the model corresponds to an increase in zero-inflation, i.e. a decrease in infection probability. Asterisks represent significance intervals: *, ** and *** refer to *P* < 0.05, *P* < 0.01 and *P* < 0.001, respectively.

*Fasciola hepatica* increased in intensity after the winter (*P*_MCMC_ < 0.001) as did strongyles, but decreased in prevalence in the summer (*P*_MCMC_ < 0.001) and prevalence was highest in the autumn compared with the winter (*P*_MCMC_ = 0.036), spring (*P*_MCMC_ = 0.042) and summer (*P*_MCMC_ < 0.001). Unlike strongyles, *F. hepatica* prevalence peaked in yearlings (*P*_MCMC_ = 0.01) rather than in calves, and decreased in prevalence and intensity thereafter. The intensity of infection was lower in 2-year-olds than in calves (*P*_MCMC_ = 0.024), and adults had lower prevalence and intensity of infection compared to all other age classes (*P*_MCMC_ = 0.002 for prevalence; *P*_MCMC_ < 0.001 for intensity). There was no evidence of a sex bias in either prevalence (*P*_MCMC_ = 0.496) or intensity (*P*_MCMC_ = 0.386) of *F. hepatica* infection, in contrast to the results of the strongyle model.

Prevalence of *E. cervi* was lower in the summer and autumn than in the winter and spring (*P*_MCMC_ < 0.001, Fig. 5), differing again from the patterns shown by either strongyles or *F. hepatica*. However, like the other parasites, *E. cervi* intensity was highest in the spring (*P*_MCMC_ < 0.001). The *E. cervi* age trend differed from the other parasites in that older age classes had higher prevalence but the lower intensity of infection. Calves showed a lower *E. cervi* prevalence than all age classes (*P*_MCMC_ < 0.001) and lower intensity compared to yearlings (*P*_MCMC_ < 0.001) and 2-year-olds (*P*_MCMC_ = 0.022). Adults had a higher prevalence than yearlings (*P*_MCMC_ = 0.044) but a lower intensity than yearlings and 2-year-olds (Both *P*_MCMC_ < 0.001). Unlike both strongyles and *F. hepatica*, *E. cervi* showed a weak female sex bias, with increased intensity in females (*P*_MCMC_ = 0.04). Prevalence was also higher in females, although this was not significant (*P*_MCMC_ = 0.15).

### Model-derived repeatability estimates

After accounting for differences in age, sex and season using overdispersed Poisson models, the individual component of variation in count differed between all parasites (*P*_MCMC_ = 0.014 for *F. hepatica* and *E. cervi*; *P*_MCMC_ < 0.001 otherwise). The direction and significance of the fixed effects from these models closely followed the Poisson component of the ZIP models. Strongyle counts had very low repeatability between-seasons with posterior mode R = 0 (95% credibility intervals 0–0.07). *F. hepatica* and *E. cervi* repeatabilities were higher: R = 0.19 (0.12–0.27) and 0.30 (0.25–0.38) respectively. However, strongyle counts were repeatable within-season, as demonstrated by variance accounted for by the ID:Season interaction term, R = 0.38 (0.24–0.49). This term was low for *F. hepatica* and *E. cervi* (R = 0 for both), showing that while both were repeatable at all levels this was expressed in the between-season individual identity term, with no additional repeatability within seasons. These results differ markedly from the raw correlations (Figs 2 and 3) as they take into account variation that is attributable to age, sex and season, thereby estimating the within-individual correlations given these factors.

## Discussion

We discovered a significant individual component of parasitism despite extensive variation in parasite counts between individuals and between seasons. Both *F. hepatica* and *E. cervi* were repeatable between seasons once age and sex category were accounted for using Poisson models, while strongyle counts were only repeatable within-season. Seasonal trends varied, with strongyles showing the most extensive seasonality, though counts of all parasites were lowest in winter. As expected younger individuals tended to have higher intensity infections than adults, but this was not mirrored in increased prevalence apart from in strongyles. Strikingly, sex biases differed between parasites, with higher strongyle intensities in males and marginally higher *E. cervi* prevalence in females. An overriding feature of this study is the contrasting and asynchronous effects shown by the different parasite taxa: despite all being helminths and having relatively similar life cycles, they exhibited substantially different seasonality and intrinsic trends as well as showing different levels of repeatability which are not attributable to their different detection assays. Studies in wild mammals which investigate multiple pathogens (e.g. Vicente *et al.* 2007*b*) often use distantly related microparasites and macroparasites with very different life cycles. Here we demonstrate the value of investigating multiple high-prevalence parasite taxa even where the chosen parasites are ostensibly similar. In accordance with previous studies (e.g. Chipeta *et al.* 2013), zero-inflated Poisson models successfully revealed that some factors affected prevalence and intensity in different directions. While factors that increased prevalence of infections also tended to increase intensity whether their effects were significant or not (Fig. 4), there were notable exceptions in the season categories: the summer (and autumn for *E. cervi*) featured increased intensity but reduced prevalence of *F. hepatica* and *E. cervi* compared with winter as a result of very low prevalence in calves. Similarly, the models distinguished between increased prevalence but reduced intensities of *E. cervi* in adults compared with younger age classes. We advocate the use of these models where sample sizes are sufficient, particularly in situations where differences between seasons and classes will result in divergent processes affecting helminth prevalence and intensity (e.g. age-related infection prepatency).

Propagule output is a function of both host and parasite biology (Sargison, 2013), representing a combination of adult worm burden and host health as well as fluctuations in worm reproduction; hence faecal propagule counts are subject to fluctuations through time. We have shown that despite this, helminth counts can be repeatable amongst wild individuals within and across seasonal time frames. In the repeated FECs strongyle egg count had a Spearman's rank correlation of *R* = 0.95, demonstrating high technical repeatability of this counting method. Within-season correlations were high for all parasites, showing that each sample taken was largely representative of an individual's parasite count regardless of when the sample was taken in the day or within the sampling trip; this also demonstrates a high reliability of the assays used and low importance for potential nuisance factors such as time to processing or time of sampling. This is in accordance with helminth studies in other ruminants (e.g. Rinaldi *et al.* 2009) – however, there may still be effects of time to collection and analysis that were not detected in this dataset and may reduce repeatability. In addition, it is noteworthy that a number individuals switched between zero and non-zero counts for all parasites (Fig. 2) and therefore a negative propagule count is not necessarily indicative of an uninfected individual, demonstrating the potential value of repeated sampling and the difficulty of diagnosing helminth infection using non-invasive faecal sampling. Similar high repeatability for different parasite taxa is nevertheless surprising, given that propagule counts were performed using three different assays which may differ in their reliability, the low intensity and possible low burden of infection and the fact that propagule shedding of all three taxa is intermittent (Gajadhar *et al.* 1994; Vercruysse and Claerebout, 2001; Schär *et al.* 2014).

Between-season correlations were lower as a result of the differences that emerged between individuals between seasons, and these correlations decreased further when repeatability was derived from Poisson models, thereby accounting for variance due to the season, age category and sex. This shows that while our counts were accurate and repeatable, much of the variation was due to certain classes (e.g. calves with strongyles) showing higher prevalence and intensity than others, and when looking within-category individuals’ counts were less consistent. Nevertheless, model-derived *R*^2^ estimates associated with individual identity did not overlap with zero for either *F. hepatica* or *E. cervi*, demonstrating consistent differences between individuals throughout the year. Strongyle counts were not as repeatable between seasons but did show individual consistency within seasons (*R*^2^ = 0.38), demonstrating that individual repeatability decreased between seasons rather than being absent at all levels. This *R*^2^ value is intermediate compared to other studies investigating strongyle FEC of wild horses within and between seasons (Wood *et al.* 2013; Debeffe *et al.* 2016) but low compared to between-season repeatability in farmed horses (Scheuerle *et al.* 2016) and goats (Hoste *et al.* 2002). Low repeatability compared with farmed animals is unsurprising given the large range of different conditions that wild individuals experience. High variability of parasite counts within individuals has implications for their quantification in wildlife. For example, an individual that shows high strongyle FEC in the spring may not do so in the summer. This demonstrates the value of multiple sampling seasons as well as reflecting the evolutionary ecology of the deer and their helminths. For example, different seasonal peaks may be associated with between-individual variation in seasonal trade-offs with immunity through e.g. reproduction (Martin *et al.* 2008) or with varying levels of tolerance to infection. Similarly, asynchronous peaks of egg output across the host population may be adaptive for the helminths in encouraging year-round transmission and bet-hedging to buffer for unfavourable climatic conditions.

All parasites showed some transmission in each sampling trip, although as the expected intensity of infection was lowest in the winter for all three parasites, with peaks in the spring and summer which continued into the autumn for *F. hepatica* and *E. cervi*. This low transmission in colder seasons likely reflects a reduction in egg production rather than solely burden, as strongyle burden in Spanish red deer stays constant or increases in the winter (Santín-Durán *et al.* 2008) and *F. hepatica* is found regularly in necropsies of the Rum deer throughout the mortality period (personal observation). Freezing, which regularly occurs on the ground in the winter on Rum, is known to damage strongyle eggs and larvae (Foreyt, 1986; Wharton and Allan, 1989) and *F. hepatica* eggs (French *et al.* 2016). However, overwinter transmission can occur in some parasite species (e.g. Carlsson *et al.* 2012); future work identifying the strongyle species present may be able to identify whether winter and autumn strongyle transmission involves a few frost-resistant species. As well as increasing the survival of environmental stages, high helminth transmission in the spring is a possible adaptive strategy resulting in coincidence between vulnerable calves and maximum infective parasites in the environment, similarly to the periparturient rise in sheep strongyle FEC (Armour, 1980). *Fasciola hepatica* and *E. cervi* showed less variation between seasons than did strongyles, which may be linked to their reduced reliance on the influx of young naïve individuals, as well as reflecting a longer lifespan of individual parasites and/or hardier propagules more capable of year-round transmission. Low seasonality of these parasites is surprising given that both go through intermediate snail hosts, their infection of which would be expected to rely on weather conditions (Olsen *et al.* 2015; Kim *et al.* 2016), and temperatures above 10 °C are required for *F. hepatica* egg development (Ollerenshaw and Smith, 1969). *Elaphostrongylus cervi* output varies according to monthly rainfall patterns in Spanish red deer (Vicente *et al.* 2005), so larval output may be dependent on environmental cues on a shorter timescale rather than fluctuating annually as do strongyles. It is important to clarify that verifying seasonal dynamics such as this would require continuous sampling rather than employing discrete seasons as we do here for practical reasons. We therefore cannot confirm exactly during which period each parasite peaks at an individual- or population-level; however, the high repeatability of *F. hepatica* and *E. cervi* between seasons supports higher population-level synchrony, with more variable strongyle transmission. Asynchrony in seasonal peaks between parasites will affect host-parasite interactions by necessitating different immune responses at different times of the year. In this case, immunity to strongyles is likely to rise in the warmer months; lower seasonality of *F. hepatica* and *E. cervi* transmission will necessitate year-round immunity to these parasites, possibly interacting with seasonal costs of variation in nutrition, mating, reproduction and maternal care.

Young individuals tended to experience higher intensity infections than adults, likely playing an important role in maintaining and transmitting helminth infections in the population. Strongyles were particularly age-biased, with calves showing a higher prevalence and intensity that decreased with each successive life stage in a similar pattern to that seen in other studies of ungulate strongyles. *F. hepatica* and *E. cervi* also closely followed previously-seen age profiles, increasing in prevalence at the yearling stage (Vicente *et al.* 2007*a*; French *et al.* 2016). However, *F. hepatica* prevalence and intensity decreased in adults while *E. cervi* increased in prevalence but decreased in intensity past the yearling stage. This age-biased infection implies that studies based on selective culling regimes that focus on, for example, adults, may indeed be missing relevant season-group categories which are important in determining the extent of parasitism within a population. For example, the high numbers of calves and yearlings sampled in our study contributed to the high prevalence of *F. hepatica* seen here (>50%) compared to that in a study of Scottish deer which largely used culled adults (French *et al.* 2016, mean 26% prevalence). The stronger age bias in strongyles may result from strongyle infection causing more mortality or more effective adaptive immunity than *F. hepatica* and especially *E. cervi*. This concurs with the view of some strongyle species as highly pathogenic (Hoberg *et al.* 2001) while *E. cervi* is often asymptomatic in red deer (Alberti *et al.* 2011). Despite having high-intensity infections in spring, and in contrast to strongyles, calves exhibited a very low prevalence of *F. hepatica* and *E. cervi* in the summer and autumn, as revealed by the zero-inflation of the models (Fig. 4). This was a likely result of infection prepatency: *F. hepatica* has an 8-week prepatent period in cattle (de León *et al.* 1981), while *E. cervi* can take 80–200 days to develop depending on dose (Gajadhar *et al.* 1994). This influence of prepatency on prevalence patterns reinforces the need to understand seasonality when investigating helminths using non-invasive methods: it is likely that many calves with zero or low counts in the summer were in fact heavily infected with both parasites, but this was not yet detectable using faecal examination. Thus, quantifying the repeatability of infection with these parasites in individual calves was not possible until the autumn (for *F. hepatica*) or the spring (for *E. cervi*).

We expected to see a male bias in all three parasites (Moore and Wilson, 2002; Zuk, 2009); however, all three parasites differed in their distribution between the sexes, with a male bias in strongyle intensity and a female bias in *E. cervi* intensity, both evident in the first year, and with no effect evident in *F. hepatica*. The male bias in strongyle infection arose late in life compared to that in Soay sheep, in which male counts greatly increase relative to female counts within months of birth (Wilson *et al.* 2004). The difference is also small (Fig. 4), and *F. hepatica* showed no sex difference, despite a previous (seasonally confounded) study showing a higher prevalence in males than females (French *et al.* 2016). On Rum most adult males live outside the study area, in areas which are at a lower density as a result of culling (Clutton-Brock *et al.* 2002) and with different geography and grazing. This may reduce exposure levels while influencing susceptibility through differences in diet. The weak female bias in *E. cervi* infection is the opposite of the expected pattern, and is particularly surprising as other red deer studies have shown male biases in *E. cervi* (Vicente *et al.* 2006, 2007*a*); opposing sex effects between parasites have been reported, e.g. in ectoparasites and endoparasites of African ground squirrels (Hillegass *et al.* 2008) and in stickleback helminths (Reimchen and Nosil, 2001), but due to a dearth of studies investigating multiple similar parasites we are not aware of any in ungulates. A possible explanation for this female bias is lower maternal care: it has been shown that hinds invest slightly less in female than male calves (Froy *et al.* 2016), and earlier weaning may result in female calves suffering higher exposure to *E. cervi*, leading to earlier patent infections, and/or they may be weaker and therefore more susceptible so show higher-intensity infections. This disparity in sex bias disagrees with the expected male bias in helminth infection and implies that male deer may in fact feature a difference in the community rather than the extent of parasitism.
